# IMG Doctors’ Experience of Starting Their First Job in the UK – Cross-Sectional Survey Study

**DOI:** 10.1192/bjo.2025.10150

**Published:** 2025-06-20

**Authors:** Marium Imran, Mahmoud Abdelaal, Raja Adnan Ahmed

**Affiliations:** 1 CWP North West Deanery, England, United Kingdom, Chester, United Kingdom; 2 CWP North West Deanery, Liverpool, United Kingdom; 3 Frenchay Brain Injury Rehabilitation Unit, Bristol, United Kingdom

## Abstract

**Aims:** To identify the difficulties International Medical Graduate (IMG) doctors face when starting their first job in the UK.

**Methods:** A survey was created on Google Forms. It included questions about the basic demographic of the IMG doctor, their experience of getting support and the difficulties they faced when they first started working in the UK. The survey had a mixture of tick boxes and free text options. The survey was distributed using social media forums and shared across IMG doctors’ social media groups. The survey link was open from December 18 to December 31, 2024. We received a total of 367 responses and 300 relevant responses were analysed, they represent the IMG doctors currently working in the UK and starting in the last 3 years.

**Results:** The 61% (185) responders were females, 35% (107) were male and the rest did not disclose their gender. Around 50% (148) were in their first year of job in the NHS while the rest were within 3 years of working in the UK. Only 7% (23) started their first job in the UK on a training grade whereas the rest had their first job as non-training grade or locally employed doctors.

76% (228) reported that they received shadowing periods as part of their first job experience and 70% (200) reported getting formal induction when they first started. 46% (125) were given access to the portfolio and 49% (134) had access to the study budget. 40% (122) did not feel they had enough senior support when they first started working in the UK. The free text boxes asked for suggestions around how the experience of IMG doctors can be improved in the UK and the responses indicated better induction as the most important factor.

**Conclusion:** The unique learning needs of the IMG doctors should be identified, and support systems should be in place accordingly. An IMG doctor-focused and tailored induction is required for the newly starting doctors. The shadowing period can also be a particularly useful tool in helping people understand the new systems and improve their confidence, but it is not offered to all new doctors.

Some IMG doctors may require enhanced supervision during the initial months, this can be delivered by peer and senior support. IMG doctors should feel welcomed in the new team as it helps to build psychological safety which can help to improve their performance.

